# *In vivo* knee kinematics of an innovative prosthesis design

**DOI:** 10.1515/med-2022-0518

**Published:** 2022-07-20

**Authors:** Michael Worlicek, Jens Schaumburger, Robert Springorum, Guenther Maderbacher, Florian Zeman, Joachim Grifka, Clemens Baier

**Affiliations:** Department of Orthopaedic Surgery, University of Regensburg, Regensburg, Germany; Department of Orthopaedic Surgery, University of Regensburg, Centre for Clinical Studies, Regensburg, Germany

**Keywords:** knee arthroplasty, knee kinematics, femoral rollback, navigation

## Abstract

Up to 20% of patients after total knee arthroplasty (TKA) are not satisfied with the result. Several designs of new implants try to rebuild natural knee kinematics. We hypothesized that an innovative implant design leads to better results concerning femoral rollback compared to an established implant design. For this pilot study, 21 patients were examined during TKA, receiving either an innovative (ATTUNE^TM^ Knee System (DePuy Inc.), *n* = 10) or an established (PFC^TM^ (DePuy Inc.), *n* = 11) knee system. All patients underwent computer navigation. Knee kinematics was assessed after implantation. Outcome measure was anterior–posterior translation between femur and tibia. We were able to demonstrate a significantly higher femoral rollback in the innovative implant group (*p* < 0.001). The mean rollback of the innovative system was 11.00 mm (95%-confidence interval [CI], 10.77–11.24), of the established system 8.12 mm (95%-CI, 7.84–8.42). This study revealed a significantly increased lateral as well as medial femoral rollback of knees with the innovative prosthesis design. Our intraoperative finding needs to be confirmed using fluoroscopic or radiographic three-dimensional matching under full-weight-bearing conditions after complete recovery from surgery.

## 1 Introduction

Total knee arthroplasty (TKA) is one of the most performed operations worldwide and is still increasing, because of its success in relieving pain and improving the function of osteoarthritic knees [1,2]. However, literature shows that TKA is associated with reduced range of motion (ROM), and sometimes even patients’ discomfort [3,4,5], finding expression in foreign body sensations, swellings, and pain. Recently, knee kinematics reached the focus of interest to influence the outcome after TKA. More physiological movement patterns correlate with better postoperative knee function [6]. Normal knee kinematics describes an asymmetrical posterior translation of the femur over the tibial base during flexion (femoral rollback) in combination with tibial internal rotation [7,8], whereas postoperative knee kinematics after TKA shows a wide range of results up to paradoxical forward slide of the femur during flexion and opposite axial rotation [9,10,11,12]. Especially posterior cruciate-retaining (PCR) knee implants seem susceptible to produce this paradoxical motion pattern, but a physiological femoral rollback is important to enable a high knee flexion [5,13,14,15]. Unphysiological knee kinematics may contribute to the still high number of unsatisfied patients [16], but there are additional reasons, which are made responsible for this. These can be patient-dependent factors like body mass index, activity level, and preoperative expectations [17,18], respectively, and also patient-independent factors, which cannot be dismissed, including surgical technique, implant position, soft tissue balancing [19], and implant design [20]. In a previous study, we could prove that our regular used knee system (PFC^TM^ Sigma cruciate-retaining, DePuy, Warsaw, IN) is able to rebuild or even improve preoperative knee kinematics, with regard to the femoral rollback [21].

The purpose of this prospective study was to analyze intraoperative knee kinematics during TKA using a computer-assisted kinematic software of an innovative PCR knee system, with regard to femoral rollback. Additionally, we compared the results to an established, regularly used PCR prosthesis. We hypothesized that an innovative implant design leads to better results concerning femoral rollback compared to an established implant design and thus improves clinical results and patient satisfaction.

## 2 Materials and methods

In this prospective study, we chose 11 patients out of a collective of 30 patients by computer randomization with primary osteoarthritis of the knee designated for TKA within a 6 months period (group 1). Patients older than 85 years of age, patients with secondary osteoarthritis of the knee, severe varus, or valgus deformity (>15°) requiring a hinged implant, or patients not willing to participate were excluded from the study.

Patients provided informed consent to this study, which was approved by the ethical committee of our Institute (Ethic Committee Approv. Number: 14-101-0326).

All 11 patients received a standard, cemented condylar prosthesis with a fixed platform, cruciate-retaining, using an innovative implant (ATTUNE^TM^, DePuy, Warsaw, IN) (group 1). These 11 patients were matched by age, sex, and preoperative leg axis with patients, who received our standard prosthesis (PFC^TM^ Sigma cruciate-retaining, DePuy, Warsaw, IN) (group 2). One patient out of group 1 had to be withdrawn, because of an intraoperative software dysfunction. This left a final data set of 21 patients. Patientscharacteristics are shown in Table 1.

**Table 1:** Demographic data of the study

Characteristics of the study group*		
	*n* = 10 (Attune)	*n* = 11 (PFC)
Gender (female) (%)	5 (50)	6 (55)
Age (years)	60.1 (SD, 9.7)	60.7 (SD, 4.6)
Treatment side (right) (%)	6 (60)	5 (45)
Leg axis (varus) (%)	9 (90)	9 (82)

*Categorical data values are given as relative and absolute frequencies; quantitative data values are given as mean with SD in parentheses.

Postoperative alignment ((1) mechanical axis) and implant position ((2) coronal femoral, (3) tibial component alignment) were measured on standing long-leg radiographs according to the Knee Society Radiological Score.


**Ethical approval:** This investigation was approved by the local Ethics Commission (No. 14-101-0326). All procedures were in accordance with the ethical standards of the responsible committee on human experimentation and with the Helsinki Declaration of 1975, as revised in 2000. All patients provided informed consent to participate in this study.

## 2.1 Surgical technique

All of the operations were performed with navigation technique (BrainLab surgical navigation system Knee, BrainLab, Feldkirchen, Germany) by two experienced knee surgeons (CB, JS) (TKA, *n* > 500) under the direction of the senior author using a standard medial parapatellar approach. Both were familiarized with the new system, before our study. After exposing the knee, two passive optical reference arrays were attached to the medial distal femur and the medial proximal tibia. After approval of the center of the hip joint by circumduction, the required anatomical land-marks were tapped. The following kinematic test included passive ROM from maximum extension to maximum flexion, during which the relative orientation between femur and tibia was displayed in real-time. No patella replacements were used.

The surgical technique was navigation-based, using a tibia first approach according to the navigation target (postoperative leg axis). Following the hospital’s standard guideline, all surgeons aimed to restore a neutral mechanical axis of the leg.

After the tibial cut, all osteophytes were removed and a medial (varus knees) or lateral (valgus knees) release was performed. Next, a tensiometer with a metric scale to match extension and flexion gap with a distraction force of 90 N was inserted and gaps were recorded. The distal femoral cut was then performed according to the navigation target. According to flexion and extension gap, the anterior and posterior femoral cuts were performed.

After implantation of the prosthesis, the kinematic test was performed. The kinematic elaboration was based on the analysis of passive ROM. For each patient, the combination of movements was registered three times.

**Figure 1:**
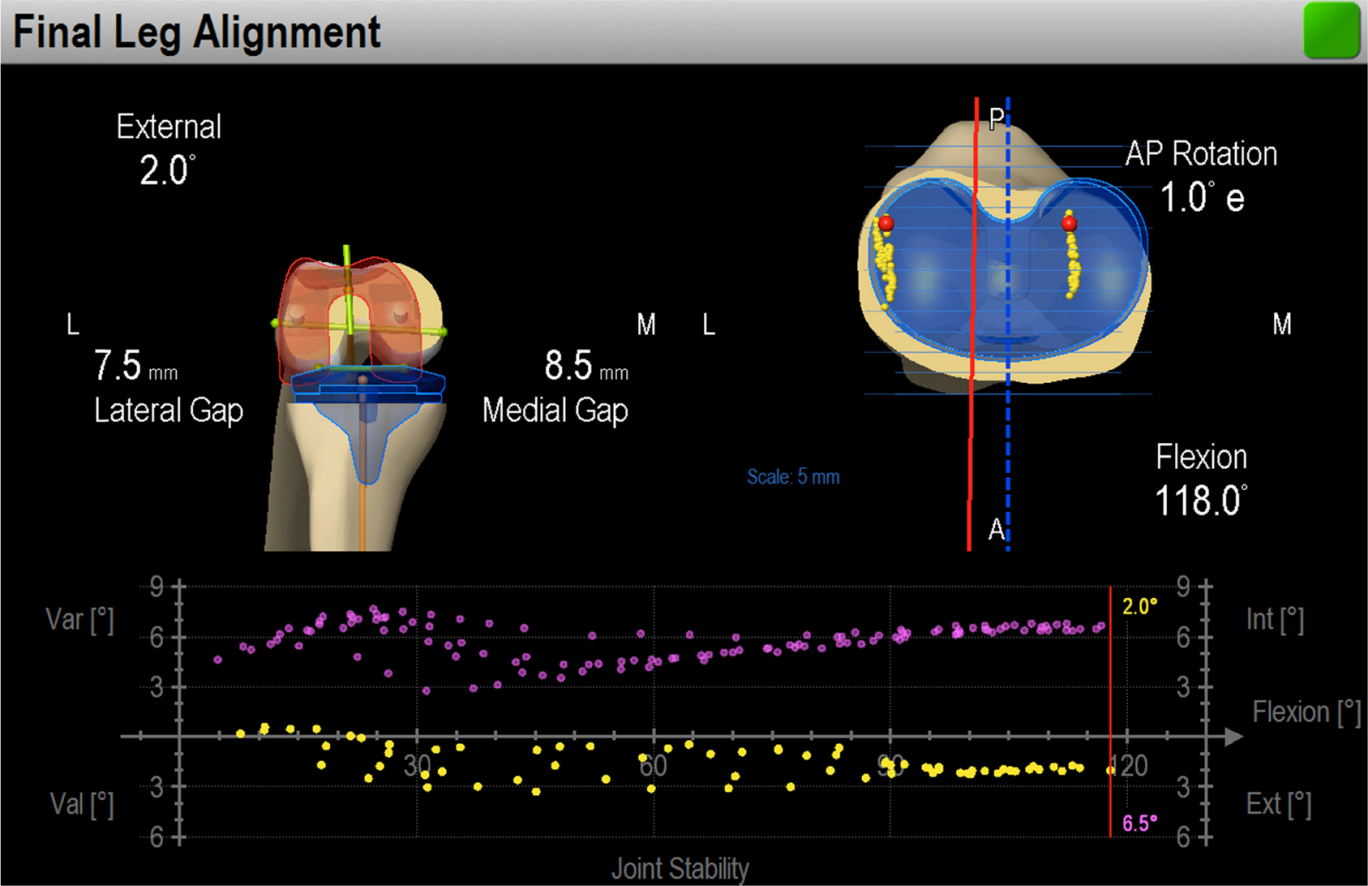
Intraoperative screenshot shows improvement of the leg axis (pink: preoperative; yellow: postoperative) and the tibial contact points of the femur during flexion (femoral rollback).

**Figure 2:**
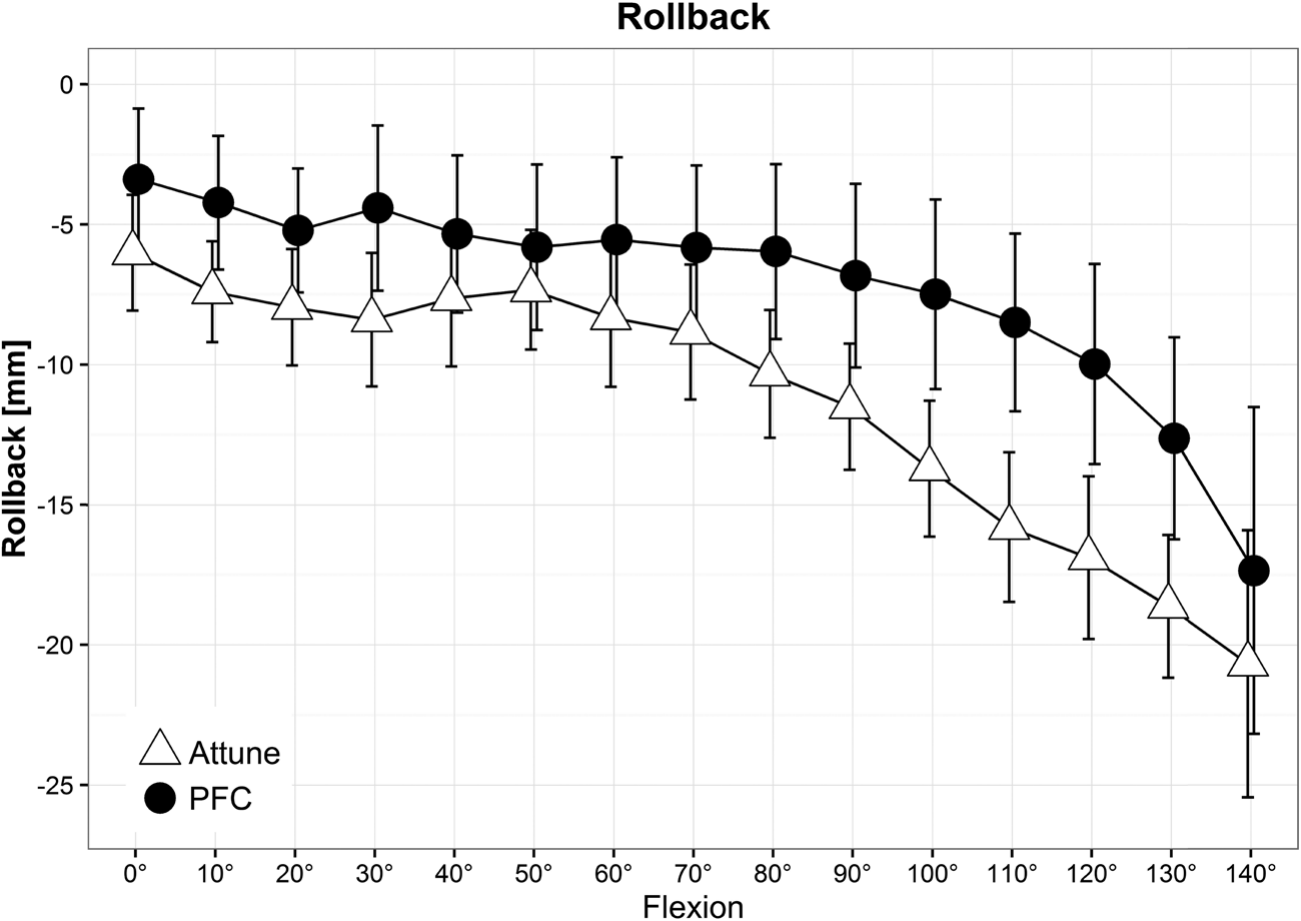
The line chart shows the mean course of femoral rollback of both groups (attune and PFC). With increasing flexion, the femoral rollback is rising, comparable to the healthy knee, with more rollback in group 1.

Intraoperative passive kinematics was measured using the BrainLab surgical navigation system (Knee 2.5.2 for PFC and Knee 2.6 for Attune; BrainLab, Feldkirchen, Germany) and analyzed by the corresponding software (Figure 1a and b). The system includes an optoelectronic localizer, two removable reference arrays (fixed on the femur and tibia using 7 mm Schanz screws), and a probe, all equipped with passive optical markers. The software allows anatomical and kinematic data acquisitions and provides real-time display of knee alignment during surgery (Figure 2) and standard kinematic evaluations.

All kinematic analyses were recorded after temporary occlusion of the joint capsule by clamping jaws. The final kinematic measurements were performed with the original components. Extension and flexion of the knee were performed by hand. The relative position of both condyles to the tibia was measured to see whether the condyles moved backward with increased flexion.

## 2.2 Statistical methods

Differences in femoral rollback between the type of prosthesis and between lateral and medial sides were analyzed using linear mixed models. Flexion was added as a random factor, and the correlation structure between the repeated measurements was set as unstructured. Results are presented as least squares means (adjusted mean values) and 95% confidence intervals (CI). All reported *p*-values are two-sided, and a *p*-value of 0.05 is considered the threshold of statistical significance. Due to the explorative nature of this study, no adjustment for multiple testing was done.

Due to the complex structure of the statistical model, an a priori sample-size calculation was not possible. It was not possible to get sufficient estimates for all parameters needed (e.g., correlation structures between flexions). Furthermore, to our best knowledge, this was a pilot study, and previous knowledge concerning measured parameters was not present.

According to Julious [22] and Hertzog [23] a sample size of 10 is sufficient for pilot studies to get first effect estimates.

Data entry and calculations were made using SAS 9.4 (SAS Institute, Cary, NC, USA). Plots were made using R 3.2.4.

## 3 Results

### 3.1 Radiological results

#### 3.1.1 Coronal alignment

The mean deviation for the mechanical axis was 1.3 ± 1.5° in group 1 and 2.1 ± 1.1° in group 2. The difference was not significant (*p* > 0.05). For the femoral frontal component alignment, the mean deviation value for group 1 was 1.8 ± 1.6° and 2.0 ± 1.6° for group 2. The difference was not significant (*p* > 0.05).

For the tibial frontal component alignment, the mean deviation value for group 1 was 1.4 ± 1.3° and 1.6 ± 1.3° for group 2. The difference was not significant (*p* > 0.05).

#### 3.1.2 Sagittal alignment

The femoral sagittal component alignment in group 1 was 3.1 ± 2.6° and 3.9 ± 3.3° in group 2. The difference was not significant (*p* > 0.05).

The tibial sagittal component alignment in group 1 was 3.7 ± 1.9° and 3.9 ± 2.1° in group 2. The difference was not significant (*p* > 0.05).

#### 3.1.3 Kinematic results

We found a significantly (*p* < 0.001) higher anterior–posterior translation in the knees of group 1 compared to group 2 during knee flexion. The mean femoral rollback over all flexions was 11.00 mm (95%-CI, 10.77–11.24) in group 1, and 8.12 mm (95%-CI, 7.84–8.42) in group 2 (Figure 2). Comparing the lateral femoral rollback between both systems, we found a mean value of 10.80 mm (95%-CI, 10.46–11.15) for group 1 and 8.07 mm (95%-CI, 7.68–8.47) for group 2 with a significant difference of 2.73 mm (*p* < 0.001).

Comparing the medial femoral rollback between both systems, we found a mean value of 11.21 mm (95%-CI, 10.57–11.39) for group 1 and 8.19 mm (95%-CI, 7.92–8.33) for group 2 with a significant difference of 3.02 mm (*p* > 0.001).

## 4 Discussion

Our study revealed a significantly increased femoral rollback over flexion of an innovative knee implant. However, medial pivoting of almost the same amount could be detected as well.

Since the introduction of TKA, over 40 years ago, there have been variations in implant designs. But there is still a high number of dissatisfied patients [8]. TKA aims to rebuild the kinematics of healthy knees, but often without success [24,25,26,27]. Several reasons for this have been discussed, but only a few can be influenced by the surgeon, like surgical technique or the use of a navigation device for higher precision in component positioning and restoration of leg alignment [19,28]. Over the last years, manufacturers have made some efforts to improve the postoperative outcome by developing new implant designs with regard to current findings of knee kinematics.

Concerning clinical outcome, literature describes an improved postoperative mobility with good stability of the innovative ATTUNE^TM^ system, compared to the common literature [29]. It was concluded that this improvement results from the consideration of natural knee kinematics and the findings of biomechanics in the last years [29].

Pre- and postoperative knee kinematics of osteoarthritic knees has shown a wide variety of results including a mostly paradoxical forward slide of the femur during flexion [9,30,31]. Normal knee kinematics describes an asymmetrical femoral rollback mechanism during flexion, predominantly of the lateral femoral condyle [7,8,32]. Using magnetic resonance imaging (MRI) technique in cadaver and living knees, Hill et al. [33] described no anteroposterior movement medially but a lateral rollback combined with sliding laterally during flexion. Measurements under weight-bearing conditions after TKA showed similar kinematic patterns of posterior stabilized and cruciate-retaining total knee arthroplasties in flexion activities [34].

Modern implant designs were, among others, developed, to improve postoperative results and patient satisfaction. One aspect was the effort to enable more physiologic movement patterns with the implant, thus a more lateral femoral rollback with tibial internal rotation during flexion.

In this study, the ROM was analyzed using fluoroscopy. Abnormal femoral translation with anterior slide of the femur during flexion is an often seen effect after TKA and might be associated with negative effects on knee kinematics and postoperative function [9,10,11,12]. In our study, both implants did not show this paradoxical movement post-implantation.

We found a significantly higher femoral rollback in group 1 with the innovative implant compared to group 2 with the established implant with increasing flexion (Figure 2). The course of motion shown in Figure 2 is comparable to anterior–posterior translation of healthy knees, which has been described in previous studies [8,33]. However, we detected medial pivoting in terms of medial femoral rollback in both implants, with the innovative implant showing a significantly increased medial pivoting. As surgical techniques, as well as patient characteristics, were consistent, the different implant designs might be a reason for the increased femoral rollback of the innovative implant.

These results are interesting, especially because to the best of our knowledge, it is so far the first study comparing intra-operative kinematics between these two implant designs using a navigation device.

Our study has several limitations. First, the number of patients seems small, but with regard to the significance level of our results, we found it adequate. Second, we did not analyze knee kinematics pre-implantation. As mentioned above, osteoarthritic knees show a high variation in knee kinematics, from almost physiological up to the paradoxical anterior slide and opposite rotation. With regard to the study of Seon et al., there seems to be a connection between pre- and postoperative knee kinematics [35]. However, this was not the emphasis of this study, but should be contemplable in future investigations. Third, we only investigated patients with stable posterior cruciate ligaments, by using cruciate-retaining technique. But it has to be mentioned that in an earlier study we did not find a significant difference in postoperative knee kinematics comparing a cruciate-retaining and a cruciate-substituting implant [21].

Fourth, we did not investigate the clinical outcome of the patients, but this was also not the emphasis of this study, but will be investigated in further studies.

Furthermore, our kinematic analysis was based on non-weight-bearing, passive motion with a lack of muscle tension. During weight-bearing, quadriceps activation plays a key role in final knee kinematics.

But as Hill et al. [33] showed in a study that analyzed tibiofemoral kinematics, there are no significant differences considering tibiofemoral movements, in the loaded and unloaded living knee. Even compared to loaded and unloaded cadaveric knees, there seems to be no significant difference in knee kinematics [28]. Likewise, Johal et al. [8] showed that femoral rollback occurs with knee flexion under loaded and unloaded conditions, but the magnitude of rollback and lateral external rotation of the femur was greater and occurred earlier in weight-bearing knees. Postolka et al. and Hill et al. [32,33] were able to show in fluoroscopic as well as MRI series that there are kinematical differences between loaded and unloaded knees. However, these differences were predictable and rather limited. Therefore, we would not expect significant differences intra- and postoperatively. So we are confident that our results are transferable to the living, weight-bearing knee. Nevertheless, the dynamic radio-stereometric analysis should be performed to clearly assess *in vivo* kinematics after TKA.

In conclusion, we could show that an innovative prosthesis design is able to improve the femoral rollback after TKA in osteoarthritic knees compared to an established system. So it can offer a more physiological course of motion and may improve the postoperative functional outcome. This, however, has to be proven in future studies including three-dimensional matching and radio-stereometric analysis under weight-bearing conditions.

## List of abbreviations

PCR: posterior cruciate retaining
ROM: range of motion
TKA: total knee arthroplasty
